# Supplementary material for: Evidence supported by Mendelian randomization: impact on inflammatory factors in knee osteoarthritis
Source: Front Med (Lausanne). 2024 May 28;11:1382836. doi: 10.3389/fmed.2024.1382836 (PMC11165061; doi:10.3389/fmed.2024.1382836)
Supplement: Supplementary file 1 [file Data_Sheet_1.ZIP › data/GCST90274812_5e6/812.leaveoneout.pdf]

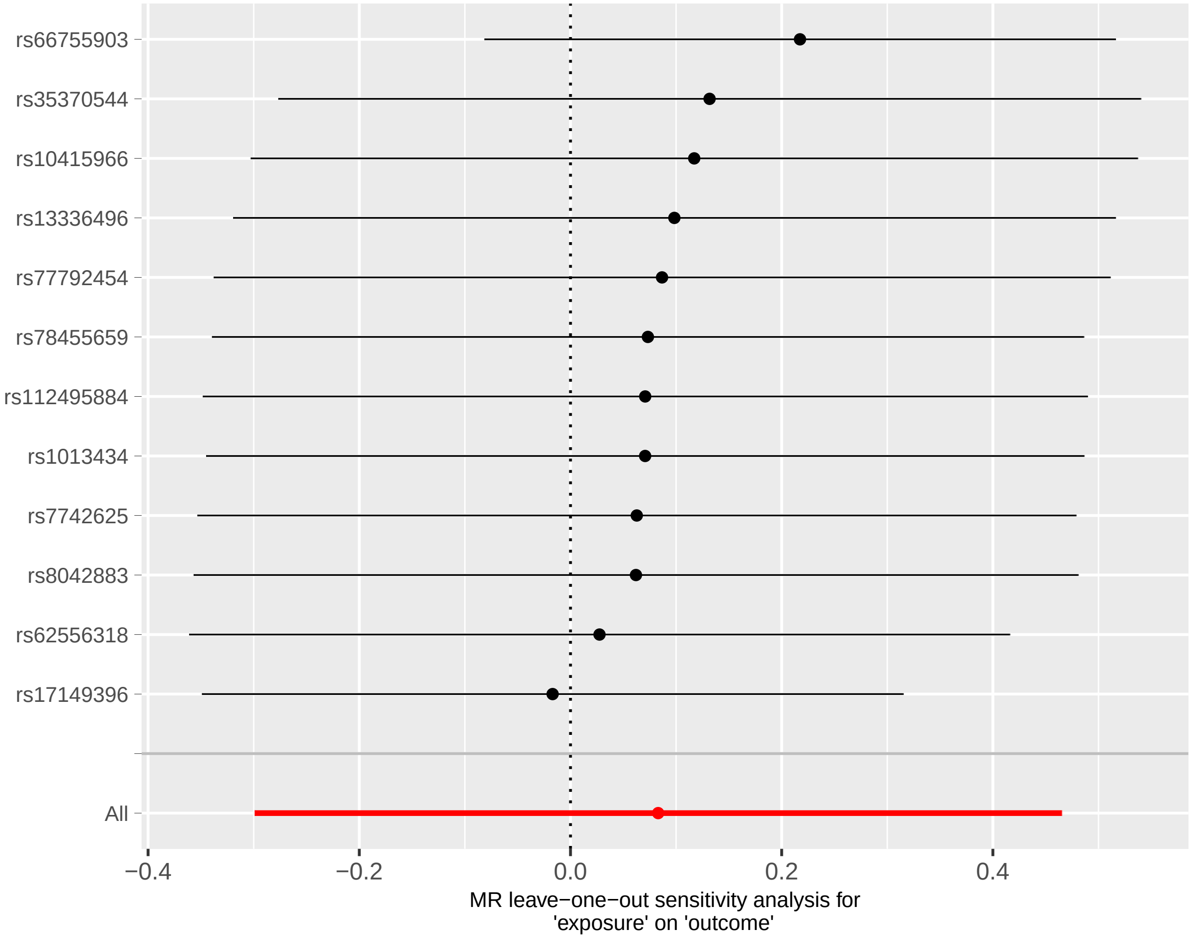
rs66755903
rs35370544
rs10415966
rs13336496
rs77792454
rs78455659
rs112495884
rs1013434
rs7742625
rs8042883
rs62556318
rs17149396
All
−0.4
−0.2
0.0
0.2
0.4
MR leave−one−out sensitivity analysis for
'exposure' on 'outcome'
